# Household food access and child malnutrition: results from the eight-country MAL-ED study

**DOI:** 10.1186/1478-7954-10-24

**Published:** 2012-12-13

**Authors:** Stephanie Psaki, Zulfiqar A Bhutta, Tahmeed Ahmed, Shamsir Ahmed, Pascal Bessong, Munirul Islam, Sushil John, Margaret Kosek, Aldo Lima, Cebisa Nesamvuni, Prakash Shrestha, Erling Svensen, Monica McGrath, Stephanie Richard, Jessica Seidman, Laura Caulfield, Mark Miller, William Checkley

**Affiliations:** 1 Fogarty International Center, National Institutes of Health, Bethesda, USA; 2 Program in Global Disease Epidemiology and Control and Division of Human Nutrition, Bloomberg School of Public Health, Johns Hopkins University, Baltimore, USA; 3 Division of Women and Child Health, Aga Khan University, Karachi, Pakistan; 4 International Centers for Diarrheal Disease Research, Matlab, Bangladesh; 5 University of Venda, Thohoyandou, South Africa; 6 Christian Medical College, Vellore, India; 7 Institute of Medicine, Kathmandu, Nepal; 8 University of Bergen, Bergen, Norway; 9 Federal University of Ceara, Fortaleza, Brazil; 10 Division of Pulmonary and Critical Care, School of Medicine, Johns Hopkins University, Suite 9121 1800 Orleans Street, 21212, Baltimore, MD, USA

## Abstract

**Background:**

Stunting results from decreased food intake, poor diet quality, and a high burden of early childhood infections, and contributes to significant morbidity and mortality worldwide. Although food insecurity is an important determinant of child nutrition, including stunting, development of universal measures has been challenging due to cumbersome nutritional questionnaires and concerns about lack of comparability across populations. We investigate the relationship between household food access, one component of food security, and indicators of nutritional status in early childhood across eight country sites.

**Methods:**

We administered a socioeconomic survey to 800 households in research sites in eight countries, including a recently validated nine-item food access insecurity questionnaire, and obtained anthropometric measurements from children aged 24 to 60 months. We used multivariable regression models to assess the relationship between household food access insecurity and anthropometry in children, and we assessed the invariance of that relationship across country sites.

**Results:**

Average age of study children was 41 months. Mean food access insecurity score (range: 0–27) was 5.8, and varied from 2.4 in Nepal to 8.3 in Pakistan. Across sites, the prevalence of stunting (42%) was much higher than the prevalence of wasting (6%). In pooled regression analyses, a 10-point increase in food access insecurity score was associated with a 0.20 SD decrease in height-for-age Z score (95% CI 0.05 to 0.34 SD; p = 0.008). A likelihood ratio test for heterogeneity revealed that this relationship was consistent across countries (p = 0.17).

**Conclusions:**

Our study provides evidence of the validity of using a simple household food access insecurity score to investigate the etiology of childhood growth faltering across diverse geographic settings. Such a measure could be used to direct interventions by identifying children at risk of illness and death related to malnutrition.

## Background

One in every five children in the developing world is malnourished, and poor nutrition is associated with half of all child deaths worldwide [1,2]. Malnutrition in early childhood can lead to cognitive and physical deficits, and may cause similar deficits in future generations as malnourished mothers give birth to low birth weight infants [3]. Malnutrition also increases susceptibility and incidence of infections and is associated with diminished response to vaccines [4]. The root of malnutrition in early childhood is complex with a variety of direct and underlying contributors related to lack of food, including insufficient breastfeeding and inadequate complementary foods; protein and nutrient loss from respiratory and gastrointestinal infections; chronic immune stimulation due to persistent parasitic intestinal infections; and inadequate water and sanitation [5,6]. Food insecurity is a key risk factor for child malnutrition [7,8]. Based on the 1996 World Food Summit, food security occurs “when all people at all times have access to sufficient, safe, nutritious food to maintain a healthy and active life” [9]. Food security comprises three hierarchical components: availability, access and utilization [10]. Availability is often measured through proxies at the population level, such as national agricultural output, while access and utilization are more often measured at the household and individual levels respectively [11]. While direct measures of food utilization exist, such as food frequency questionnaires [12], household food access has often been measured indirectly, through child anthropometry [10] or agricultural productivity [12]. Measurement of all three aspects of food insecurity has posed persistent challenges, such as the difficulty in measuring the impact of short-term shocks on household food access [12]. Recent research, however, shows promise in the area of food access measurement, with the construction of simple household survey measures such as the Household Food Insecurity Access Scale (HFIAS) [11,13,14]. Low-cost and valid measures of household food insecurity are necessary to accurately predict the prevalence of food insecurity in response to changing conditions [15]. Such measurements can then inform targeted interventions to diminish childhood morbidity and mortality [10,12]. However, global progress against food insecurity requires measures that are valid and comparable across countries. We sought to assess the acceptability, validity, and generalizability of the HFIAS, an existing nine-item measure of household food access, in the setting of a multi-country study. To achieve this aim, we collected cross-sectional data on household food access insecurity and child nutritional status, as measured by anthropometry, in eight country sites to determine whether these variables were related, and whether this relationship was consistent across diverse populations.

## Materials and methods

### Study setting

We conducted our study at the eight field sites in the Malnutrition and Enteric Infections: Consequences for Child Health and Development (MAL-ED) Network cohort study. The MAL-ED Network, comprising researchers from thirteen academic and research institutions, aims to explore the relationship between malnutrition and intestinal infections and their consequences for various aspects of child growth and development. Sites are utilizing a standardized protocol for the collection of twice-weekly diarrhea surveillance information, monthly anthropometry, urine for gut function and iodine status, stool for enteric pathogens, blood for micronutrients and vaccine response, and cognitive development assessments. Study sites are located in rural, urban, and peri-urban areas of Bangladesh, Brazil, India, Nepal, Pakistan, Peru, South Africa and Tanzania (See Additional file 1). The MAL-ED study began enrolling pregnant women in 2009, and plans to follow a cohort of approximately 200 newborns per site for up to 36 months. We report on pilot study activities that preceded enrollment for the cohort study, aimed at characterizing the relationship between food access and child nutritional status.

### Study design

In preparation for the MAL-ED cohort study, we sought to develop and test cross-country indicators of food access insecurity and socioeconomic status (SES). We administered a standardized survey including demographic, SES, and food access questions to 100 households in each of the eight field sites between September 2009 and August 2010. Households were randomly selected from census results collected within the previous year at each study site. Households were eligible to participate if they were located within the MAL-ED study area and had an index child aged 24 to 60 months. Data collection lasted approximately two to four weeks in each site. We obtained ethical approval from the Institutional Review Boards at each of the participating research sites, at the Johns Hopkins Bloomberg School of Public Health (Baltimore, USA) and at the University of Virginia School of Medicine (Charlottesville, USA). Demographic and SES questions were adapted from the most recent Demographic and Health Surveys [16] in collaboration with site investigators. Questions focused on age and education of the head of household and child’s mother, as well as the mother’s fertility history. The SES section included a series of questions on household assets, housing materials, and water and sanitation facilities. The questionnaire was developed in English, and then translated into local languages by site investigators using appropriate local terms (See Additional file 2). The questionnaire was accompanied by standard operating procedures based on existing guidelines for administration of the HFIAS [17]. Field supervisors trained field workers prior to survey administration, and used locally appropriate management techniques to support complete, accurate and timely data collection, including weekly review of all data to ensure quality.

### Food access insecurity score

To assess food access insecurity, our survey included the nine-question HFIAS (See Online Supplement), adapted in 2006 by the Food And Nutrition Technical Assistance (FANTA) project for use in low resource settings [18]. Although this scale has been validated and adapted in individual country settings through previous studies [18-20], to our knowledge it has not been used in its original form in a multi-country study. The nine-item scale uses a four-week recall period and captures three dimensions of the access component of household food insecurity: anxiety and uncertainty about household food access (item 1); insufficient quality (items 2–4); and insufficient food intake and its physical consequences (items 5–9) [18]. Responses on the nine items were summed to create the food access insecurity score, with a minimum score of 0 indicating the most food access secure households, and a maximum score of 27 indicating the most food access insecure households. We also categorized households into four groups [17]: food access secure, and mildly, moderately and severely food access insecure.

### Anthropometry

We measured height and weight in one child aged 24 to 60 months in each participating household. When multiple children in this age range lived in one household, we randomly chose one child to avoid intra-household correlation in our data. Trained field staff measured standing height to the nearest 0.1 cm using a locally produced platform with sliding headboard. Digital scales were used to measure weight to the nearest 100 grams. Height-for-age (HAZ) and weight-for-height (WHZ) Z-scores were calculated based on World Health Organization child growth standards [21]. We defined stunting and wasting as a HAZ and WHZ that were two standard deviations below the WHO standard, respectively.

### Biostatistical methods

Exploratory analyses involved examination of the distribution of each variable and inter-relationships between variables within and across sites. We then conducted a series of pooled analyses, including data from all eight country sites. We used a generalized additive model with a smoothing spline to characterize the relationship between food access insecurity and nutritional indicators. Our findings indicated that the pooled relationship between food access insecurity and both nutritional indicators was approximately linear, indicating the appropriateness of linear regression models. We then examined bivariate relationships between food access insecurity, HAZ, WHZ and SES indicators. Last, we used linear regression to model the relationship between food access insecurity and each nutritional outcome in the pooled sample of households, adjusted for child age, sex, maternal education, household bank account, people per room in the household, and access to an improved water source and sanitation facilities. We selected these SES indicators based on their relevance to the outcomes and sufficient variation within each country site. We compared the results to a model including a household SES score generated through principal components analysis based on 17 indicators of household wealth. The results were similar, and we felt that the selection of individual indicators provided more interpretable information on the relationships between food access insecurity and SES. To control for differences in baseline levels of HAZ and WHZ, we included indicator variables for all but one country. We conducted a likelihood ratio test comparing a full model with interactions between food access insecurity score and the eight country dummy variables with a reduced model lacking those interactions. The results of this test provided evidence of the extent of heterogeneity in the relationship between food access insecurity and HAZ across countries. We used R (http://www.r-project.org) and STATA 12 (STATA Corp., College Station, USA) for statistical analysis.

## Results

### Characteristics of study populations

We surveyed a total of 800 households. One child had missing anthropometry and ten had extreme anthropometric values (greater than six standard deviations from the mean) based on the WHO standard [21]. This resulted in a final sample size of 789 households (98.6% of original sample). The mean age of sampled children was 41 months (SD = 10.4); 51.5% of children were male, ranging from 58.6% in Tanzania to 44.3% in Pakistan. Variation in household SES across country sites was evidenced by variations in maternal education (3.3 years in Pakistan to 10.1 years in South Africa) and proportion with a bank account (2% in India to 76% in South Africa) (Table 1). Furthermore, the mean household SES score, calculated through principal components analysis, ranged from a low of −2.30 in Tanzania to high of 2.08 and 2.16 in Brazil and South Africa, respectively (See Additional file 1). Nearly all households, with the exception of those in Tanzania, had access to electricity and reported access to improved water and sanitation, as defined by the World Health Organization [22].

**Table 1:** Selected household characteristics overall and by country (n = 789)

		**Overall**	**Bangladesh**	**Brazil**	**India**	**Nepal**	**Pakistan**	**Peru**	**South Africa**	**Tanzania**
	**Sample size**	**789**	**99**	**98**	**100**	**100**	**98**	**99**	**96**	**99**
SES Indicators	Owns bank account (%)	31	23	21	10	62	39	15	76	2
	People per room (mean)*	1.7	3.7	1.3	3.9	2.5	5.5	1.6	1.2	1.7
	Mean maternal education (years)	6.4	3.7	7.8	6.7	6.6	3.3	7.8	10.1	5.3
	Owns Mattress (%)	58	66	98	1	99	13	82	66	39
	Owns mobile phone (%)	68	63	81	53	96	68	31	96	54
	Owns radio or transistor (%)	41	11	74	2	48	12	55	82	46
	Has electricity (%)	84	100	99	97	99	98	85	94	0
	Owns table (%)	57	29	86	21	65	50	100	74	33
Hygiene Indicators	Improved water source (%)	86	100	100	100	98	100	98	65	28
	Improved sanitation facility (%)	72	100	100	37	100	74	84	84	1
Food Access Insecurity Categories^§^	Food secure (%)	37.5	33.3	32.7	30.0	73.0	22.5	20.2	20.8	66.7
	Mildly insecure (%)	11.4	15.2	9.2	5.0	7.0	12.2	27.3	9.4	6.1
	Moderately insecure (%)	27.5	33.3	11.2	29.0	12.0	48.0	29.3	40.6	17.2
	Severely insecure (%)	23.6	18.2	46.9	36.0	8.0	17.4	23.2	29.2	10.1

*People per room is the number of people who usually sleep in the house divided by the number of rooms in the house that are used for sleeping.

^§^Food access insecurity categories are based on the guidelines in Coates et al. 2007.

### Household food access insecurity scores

**Figure 1:**
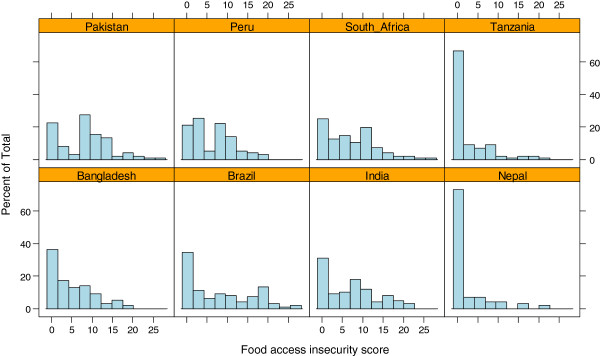
Barplots of food access insecurity score by country; 2009–10.

Food access insecurity score distributions were skewed right, indicating a large subgroup of households reporting no food access insecure experiences in the preceding four weeks (Figure 1). Across sites, 37% of all households reported no food access insecurity in the last four weeks (score of 0). This value ranged from 18% of households in Peru to 72% in Nepal. Nepal (2.4) and Tanzania (2.6) had the lowest mean scores, as well as the smallest variability between households (SD = 4.8 for both), while Pakistan (8.3) and Brazil (7.9) had the highest mean scores. Nearly half (46.9%) of households in the Brazilian site reported severe food access insecurity, whereas the majority of households in Nepal (73.0%) and Tanzania (66.7%) indicated food access security.

### Nutritional indicators

**Figure 2:**
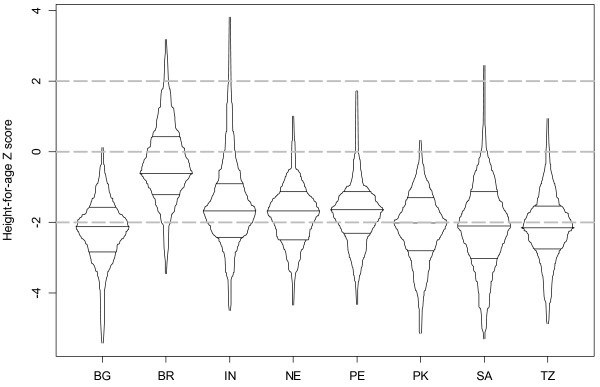
Box-percentile plots of height-for-age (HAZ) by country; 2009–10.

**Figure 3:**
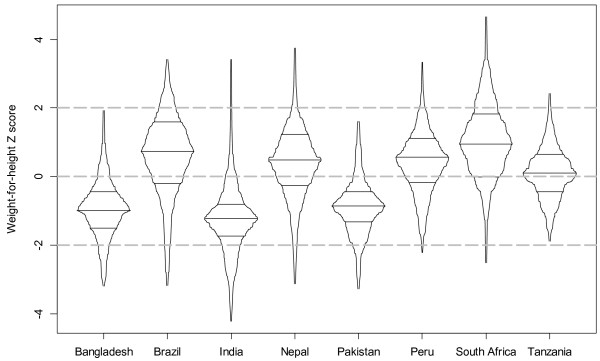
Box-percentile plots of weight-for-height (WHZ) by country; 2009–10.

Overall, 42% (ranging from 8% to 55%) of children were stunted, and 6% (range from 0% to 17%) were wasted (Figures 2 and 3). HAZ in India and Brazil were shifted toward the highest values, with approximately 35% of Brazilian children and 30% of Indian children measuring above the WHO standard mean. In the remaining six sites, approximately 50% of each population was stunted, and in Bangladesh all children were below the WHO standard mean in height. On average, a much smaller proportion of children in these sites experienced growth faltering as assessed by WHZ. In both South Africa and Tanzania, where over 50% of the sample children were stunted, none of them were wasted. In contrast, in India, where about 22% of children were stunted (fewer than most sites), a similar proportion (17%) were wasted (more than most sites). Stunting was significantly associated with infant age, water source, sanitation facility, mother’s education, and people per room. Wasting was associated with water source and people per room. Low food access security was significantly associated with sex of the child, mother’s education, ownership of a bank account, and people per room. Wasting and stunting were only weakly correlated with each other (*r* = −0.02; p < 0.001), but stunting was directly associated with inadequate water and sanitation facilities (Table 2). To further explore these relationships, we controlled for the same set of SES indicators in our regression models (Table 3). The final models for the relationship between food access insecurity and child malnutrition (HAZ and WHZ) retained the SES indicators that remained statistically significant, i.e. water source, mother’s education, and people per room. This model was more parsimonious, and the relationship of interest remained consistent between models.

**Table 2:** Relationship between socioeconomic status and nutritional indicators

	**N**	**% Stunted (HAZ < −2)**	**p-value **^**†**^	**% Wasted (WHZ < −2)**	**p-value **^**†**^	**% Severely food access insecure**^**§**^	**p-value **^**†**^
Sex							
Male	406	42.1	0.95	6.7	0.50	19.5	**0.005**
Female	382	41.9		5.5		28.0	
Age							
24-35 months	284	41.2	**0.01**	5.3	0.07	19.3	0.06
36-47 months	243	49.0		4.1		23.9	
48-60 months	262	36.3		8.8		27.9	
Water Source							
Not improved	109	58.7	**<0.001**	0.0	**<0.01**	24.8	0.75
Improved	680	39.3		7.1		23.4	
Sanitation Facility							
Not improved	218	49.5	**<0.01**	6.4	0.81	25.7	0.39
Improved	571	39.1		6.0		22.8	
Maternal education							
None	135	57.0	**<0.001**	5.2	0.15	22.2	0.59
1-5 years	174	43.1		9.1		26.4	
>5 years	480	37.3		5.2		22.9	
Bank Account							
No	545	42.2	0.83	6.6	0.36	28.3	**<0.0001**
Yes	244	41.4		4.9		13.1	
People per room							
<2	433	35.3	**<0.001**	10.1	**<0.001**	20.0	**0.023**
≥2	356	50.0		2.8		26.9	

^§^Severe food access insecurity is defined based on the guidance in Coates et al. 2007.

^†^p-values reflect results of t-tests and one-way ANOVA tests.

**Table 3:** Final models exploring the relationship between food access insecurity score and two measures of growth faltering, controlling for indicators of SES

	**Height-for-age**		**Weight-for-height**	
	**Full model**	**Final model**	**Full model**	**Final model**
Intercept (Tanzania as reference)	−1.96 (<0.001)	−2.20 (<0.001)	0.71 (0.003)	0.51 (0.007)
Bangladesh	−0.09 (0.73)	−0.14 (0.53)	−1.02 (<0.001)	−1.04 (<0.001)
Brazil	1.57 (<0.001)	1.52 (<0.001)	0.56 (0.02)	0.55 (<0.001)
Peru	0.16 (0.50)	0.14 (0.51)	0.30 (0.19)	0.27 0.09)
India	0.50 (0.03)	0.48 (0.03)	−1.26 (<0.001)	−1.37 (<0.001)
Pakistan	0.18 (0.48)	0.14 (0.56)	−0.97 (<0.001)	−1.07 (<0.001)
Nepal	0.18 (0.47)	0.12 (0.55)	0.33 (0.17)	0.28 (0.06)
South Africa	−0.10 (0.68)	−0.16 (0.40)	0.88 (<0.001)	0.85 (<0.001)
**Food access insecurity score (effect per unit score)**	**−0.020 (0.009)**	**−0.020 (0.008)**	**0.011 (0.13)**	**0.010 (0.13)**
Age	−0.005 (0.24)		−0.01 (0.005)	−0.01 (0.004)
Sex	−0.03 (0.71)		−0.08 (0.30)	
Water Source^‡^	0.38 (0.03)	0.37 (0.03)	−0.16 (0.33)	
Sanitation Facility^‡^	−0.03 (0.81)		0.12 (0.35)	
Maternal education (years)	0.02 (0.06)	0.02 (0.06)	−0.005 (0.69)	
Bank account	−0.06 (0.57)		−0.05 (0.62)	
People per room	−0.06 (0.03)	−0.06 (0.03)	−0.02 (0.53)	
Adjusted R^2^	20.3%	20.6%	35.1%	35.2%

Rows contain effect estimates and p-values in parentheses.

^*‡*^*Dichotomous variables measuring access to improved facilities based on WHO standards.*

### Association between food access insecurity and nutritional indicators

**Figure 4:**
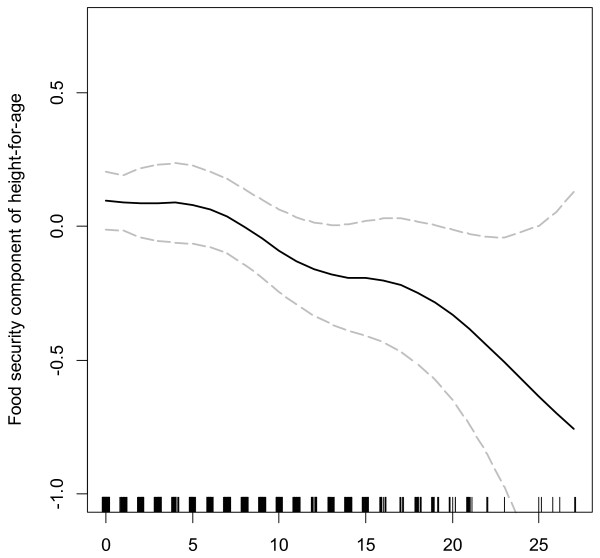
**Relationship between food access insecurity score and height-for-age (HAZ); 2009–10.** We fitted a smoothing spline to study the relationship between food access insecurity score and HAZ using a generalized additive model. The figure shows the fitted smoothing spline and corresponding 95% confidence intervals.

In exploratory analyses, the relationship between food access insecurity and HAZ was approximately linear (Figure 4). Food access insecurity score was statistically significantly associated with HAZ (p = 0.008), but not with WHZ (Table 3). In pooled regression analyses, a 10-point increase in food access insecurity score was associated with a 0.20 SD decrease in HAZ score (95% CI 0.05 to 0.34), controlling for water source, maternal education and people per room. Sensitivity analyses indicated that the use of individual indicators of SES and the use of a linear combination of indicators using principal components analysis produce similar results with respect to our research question (results not presented). We chose to include individual SES indicators in our model for ease of interpretation. A likelihood ratio test comparing nested models with and without interactions terms indicated that the relationship between food access insecurity score and HAZ did not vary significantly across countries (p = 0.17). Moreover, none of the individual interaction terms between food insecurity and site achieved statistical significance at the 0.05 level (See Additional file 1 and Additional file 2).

## Discussion

In this study, we found that food access insecurity was associated with a statistically significant shift in the distribution of children’s HAZ toward lower values, after adjusting for sociodemographic factors. Although prevalence of both food access insecurity and faltering in HAZ varied across countries, a likelihood ratio test for heterogeneity revealed that the relationship between these variables was consistent across countries. Our findings on the epidemiology of growth faltering are consistent with the literature. Previous studies have reported higher prevalence of stunting than wasting within populations [23-25], and more variation in wasting than in stunting across populations [1,25]. Although our results indicate regional patterns in prevalence of wasting only, others have found clear regional patterns in both stunting and wasting [1,23,25]. Variations between sites likely reflect the impact of numerous factors, including seasonal effects on the food supply, patterns of enteric infections, genetic predispositions, and access to prenatal and infant health services. Stunting and wasting are indicators of chronic and acute malnutrition, respectively [11]. However, beyond reflecting differences in the length of exposure to deprivation, they are also differentially associated with other socio-demographic variables, such as maternal education and immunizations [23,25]. Given different risk factors for wasting and stunting, and the weak correlation between these measures in our data, it is not surprising that food access insecurity was associated with faltering in HAZ but not WHZ. In addition to different risk factors, growth faltering in WHZ tends to occur at younger ages and result in higher mortality than faltering in HAZ [1]. Given the age of children enrolled in this study (older than 24 months), they were more likely to be stunted or healthy than to be wasted. Further research is warranted on approaches to expanding this household food access insecurity measure to more effectively capture factors associated with wasting. Patterns in SES, food access insecurity, and growth faltering were not clearly clustered by region, and no country ranked consistently highest or lowest in all factors. For example, Tanzanian households were among the poorest when measured by socioeconomic indicators, but were also among the most food access secure. We hypothesize that this difference in rankings by food access insecurity and household SES might be due to the predominantly agricultural setting, where reporting bias on food access insecurity might be more common, and where wealth may not be as closely tied to food access security as in urban settings. The opposite pattern was true of Brazilian households, which also had among the highest mean values of HAZ and WHZ scores. Our results indicate that food access insecurity was not simply an indicator of SES, but was also independently associated with growth faltering. The effect of a five-point decrease in food access insecurity was roughly comparable to the effect of a five-year increase in mother’s education on HAZ, and was approximately equal to one-third the effect of access to an improved water source. Although our analyses reveal that food access insecurity is independent of these socio-demographic indicators, these relationships warrant further exploration. The complexity of these relationships underlines the utility of a simple measure, such as the HFIAS, that could potentially predict growth faltering in children. The Food And Nutrition Technical Assistance (FANTA) project has worked since 2000 to validate and adapt the HFIAS [18]. Recent validation work in multiple countries has produced mixed results, leading investigators to suggest a shortened version of the scale, called the Household Hunger Scale, comprising only the final three items related to hunger [26]. The adapted version of the scale did not achieve statistical significance, suggesting that the full scale may be a better measure of chronic malnutrition, or that these two scales capture different information. However, in the context of the MAL-ED study, the full scale is more appropriate than the reduced scale for two reasons. First, more items generally result in higher scale reliability [27]. Second, we seek to measure the full experience of food access insecurity to facilitate exploration of the relationships between food access, food utilization, enteric infections, and nutritional markers in the early years of life. These results also provide evidence of the acceptability and validity of the nine-item HFIAS in a multi-country research setting. We were able to use the questions in their original form (with translation) in diverse cultural settings with limited problems in administration and no missing data. Our results, demonstrating a statistically significant relationship between food access insecurity and HAZ – two variables that we would expect to be correlated – provide evidence of the construct validity of the HFIAS scale in a multi-country setting [28]. Furthermore, although this measure only focuses on the access aspect of food insecurity, previous research has indicated that it correlates with dietary quality and the intake of a micronutrient rich diet, two aspects of food utilization [20]. Finally, the lack of heterogeneity in this relationship across countries provides evidence of generalizability of its use in diverse low-income settings. The MAL-ED cohort study will allow us to look at food utilization and its relationship with food access more closely through inclusion of longitudinal measures of dietary intake and repeated measurement of food access insecurity. Our study has some potential limitations. The data are cross-sectional, preventing the collection of important longitudinal risk factors for malnutrition, such as intestinal infections. However, the statistically significant association between food access insecurity and HAZ indicates the utility of a short food security survey to screen for chronic malnutrition in settings where other data are not available. Our pilot study included children aged 24 to 60 months, although wasting effects are often greatest in the first two years of life [1]. The MAL-ED cohort study will follow children from birth, collecting data on diarrheal incidence and infectious agents, seasonal changes in food access insecurity, and other important exposures, such as dietary intake. In addition, some MAL-ED study sites raised concerns that responses to certain food access insecurity items might be culturally dependent, as has been shown by Coates and colleagues [14]. For example, although researchers in the Pakistan site felt that the HFIAS was robust to concerns, they noted the potential for bias given cultural stigma against reporting food insecurity. These differences are particularly relevant with regard to selecting universal cut points for food access insecurity, rather than associations between the continuous measure and outcomes. While further inquiry is warranted on cross-country variations in response thresholds, previous research indicates that the domains of food access insecurity that form the basis of the nine-item scale are similar across cultural settings (i.e. insufficient quantity, inadequate quality, and uncertainty or worry) [14]. Also, our pilot study was not designed to assess the important role of seasonality in household food access insecurity (Additional file 1 and Additional file 2); however, we are assessing seasonality in the MAL-ED cohort study, in which we are measuring food access insecurity every six months based on child enrollment. Finally, factors affecting child growth are present not only at the individual and household levels but also at the community, national, and regional levels. Information provided through a household survey can only explain a limited amount of variation in child growth outcomes [29]. In summary, a simple household food access insecurity score can help explain differences in HAZ distributions in a multi-country study, even after adjustment for demographic and SES indicators, and country-level differences. While we do not suggest that this tool should replace the collection of child anthropometry to assess nutritional status, it could be used as a rapid assessment tool to identify households at risk of child growth faltering. Given the simplicity of this measure, and its acceptability and validity in cross-country settings, we advocate its inclusion in research and programs seeking to understand and ameliorate the predictors of child malnutrition in developing countries.

## Competing interests

The authors declare that they have no competing interests.

## Authors’ contributions

SP and WC contributed equally to the conception, design and analysis of data, interpretation of findings, and writing of manuscript. ZB participated in study conception, design and data acquisition, and a critical review of the manuscript. JS, SR, MMc, LC, MMi participated in study design and critical review of the manuscript. TA, SA, PB, MI, SJ, MK, AL, CN, PS, and ES contributed to study design and data acquisition. WC had ultimate oversight over the study design, data analysis and writing of this manuscript. All authors read and approved the final manuscript.

## Supplementary Material

Additional file 1: Table S1 Description of MAL-ED Study Sites, **Table S2.** Household Food Insecurity Access Scale, **Table S3.** Regression Results for the Household Hunger Scale vs. HAZ, **Table S4.** Season of food insecurity data collection, **Table S5.** Description of food supplementation programs in study community. Click here for file

Additional file 2 Translations of food insecurity questionnaire into Portuguese, Spanish, Sindhi, Swahili and Nepali. Click here for file
