# Supplementary material for: Household food access and child malnutrition: results from the eight-country MAL-ED study
Source: Popul Health Metr. 2012 Dec 13;10:24. doi: 10.1186/1478-7954-10-24 (PMC3584951; doi:10.1186/1478-7954-10-24)
Supplement: Additional file 1: Table S1 — Description of MAL-ED Study Sites, Table S2. Household Food Insecurity Access Scale, Table S3. Regression Results for the Household Hunger Scale vs. HAZ, Table S4. Season of food insecurity data collection, Table S5. Description of food supplementation programs in study community. [file 1478-7954-10-24-S1.doc]

Supplemental Table 1. Description of MAL-ED Study Sites

| **Country** | **Site Description** | **Mean Food Access Insecurity Score (SD)** | **Mean SES Score (SD)** |
| --- | --- | --- | --- |
| Bangladesh | Mirpur, an under-privileged community in Dhaka, is inhabited by poor and middle class families. Residential and sanitary conditions are typical of any congested urban settlement. The investigators have ongoing research activities in the area. | 4.90 (5.12) | -1.38 (1.49) |
| Brazil | Parque Universitário is an urban community inhabited by poor and middle class families. The community has approximately thirty three thousand people with 12% less than five years old. Of 288 children ≤3 years old, 31% have <-1 and 9% <-2 HAZ. | 7.90 (7.79) | 2.08 (1.33) |
| India | The study site is situated in a slum area in Vellore which is a small city in Tamil Nadu in southern India. It is predominantly inhabited by poor families. The major occupation is manual labour in the market or construction work. | 7.03 (6.34) | -1.86 (1.13) |
| Nepal | The study site Bhaktapur Municipality and adjoining villages are semi urban area, with facility for safe drinking water and toilet facilities. Main economical base is agriculture. | 2.36 (4.76) | 1.57 (1.89) |
| Peru | The site is peri-urban with an economic base in agriculture, extraction of forest products, and fishing. | 6.26 (5.11) | 0.64 (1.36) |
| Pakistan | The Molhan study site in district Naushahro Feroze is situated in southern Sindh province of Pakistan. The panorama is fertile plains near Indus river with predominantly rural communities, agricultural occupations, low socioeconomic class and poor infrastructure characterized by mud houses. | 8.28 (6.20) | -0.82 (1.89) |
| South Africa | The Rambuda site is rural and mountainous, characterized by agricultural livelihood, low socio-economic status, poor infrastructure with waterfalls and many rivers across the villages. It is situated 25 km from the central business district. | 7.16 (6.20) | 2.16 (1.70) |
| Tanzania | The Haydom area is ethnically and geographically diverse, situated at approximately 1700 meters above sea level, and 300 kilometers from the nearest urban center. The study population is mainly agro-pastoralists. | 2.62 (4.80) | -2.30 (1.32) |
|  | OVERALL | 5.80 (6.22) | 0.00 (2.29) |

*SES score is based on principal components analysis of 17 indicators of household wealth

**Supplemental Table 2. Household Food Insecurity Access Scale.**

| In the past four weeks: | |
| --- | --- |
| 1 | Did you worry that your household would not have enough food? |
| 2 | Were you or any household member not able to eat the kinds of foods you preferred because of a lack of resources? |
| 3 | Did you or any household member have to eat a limited variety of foods due to a lack of resources? |
| 4 | Did you or any household member have to eat some foods that you really did not want to eat because of a lack of resources to obtain other types of food? |
| 5 | Did you or any household member have to eat a smaller meal than you felt you needed because there was not enough food? |
| 6 | Did you or any other household member have to eat fewer meals in a day because there was not enough food? |
| 7 | Was there ever no food to eat of any kind in your household because of lack of resources to get food? |
| 8 | Did you or any household member go to sleep at night hungry because there was not enough food? |
| 9 | Did you or any household member go a whole day and night without eating anything because there was not enough food? |

*Each question has the following response categories: No (0), Rarely (1), Sometimes (2) Often (3).*

*The answers are summed to form a scale with values from 0 to 27.* *Reference: (18)*

**Supplemental Table 3. Regression Results for the Household Hunger Scale vs. HAZ**

|  | Height-for-Age Z Score (HAZ) |
| --- | --- |
|  | Hunger Only Model* |
| Intercept (Tanzania as reference) | -2.79 (<0.001) |
| Household Hunger score (HHS) | **0.046 (0.10)** |
| Bangladesh | -0.16 (0.46) |
| Brazil | 1.46 (<0.001) |
| Peru | 0.07 (0.78) |
| India | 0.45 (0.04) |
| Pakistan | 0.06 (0.78) |
| Nepal | 0.12 (0.56) |
| South Africa | -0.24 (0.20) |
| HFIAS*Bangladesh |  |
| HFIAS*Brazil |  |
| HFIAS*Peru |  |
| HFIAS*India |  |
| HFIAS*Pakistan |  |
| HFIAS*Nepal |  |
| HFIAS*South Africa |  |
| Water Source^‡^ | 0.39 (0.03) |
| Maternal education (years) | 0.02 (0.05) |
| People per room | -0.07 (0.009) |
| Adjusted R^2^ |  |

* Household Hunger Scale (last three items of HFIAS) is the outcome

**Supplemental Table 4. Season of food insecurity data collection**

| **Country** | **Description of timing of data collection** |
| --- | --- |
| Bangladesh | Data were collected during the post monsoon season from August to September of 2009. |
| Brazil | Fortaleza area has basically two seasons, called rainy and dry seasons with temperature range from 25-35 degrees Celsius with high humid all year long. The data were collected during the dry season from 7-10 June of 2010. |
| India | Data were collected August to September 2009 in the post monsoon season. |
| Nepal | Data collection and the four-week recall period took place during March of 2010, three months after the harvesting of the main crop, rice. |
| Peru | Data collection was done during the time of year when fish are plentiful, but there are no drastic changes in food security throughout the year. |
| Pakistan | Naushahro Feroze lies in a [tropical](http://en.wikipedia.org/wiki/Tropical) to [subtropical](http://en.wikipedia.org/wiki/Subtropical) region; typically, summer is characterized by hot dry weather and temperatures frequently rise above 46 [°C](http://en.wikipedia.org/wiki/Celsius) between May and August. The data collection took place from 17^th^ to 24^th^ September 2009 and the weather was mildly hot, sunny and dry. There were no rains in the month prior to data collection. |
| South Africa | Data were collected in January of 2010, which is a rainy season characterized by vegetation growth. The four-week recall period included the festive season (i.e. Christmas and the holidays with high spending). |
| Tanzania | The data were collected over a period of one month from November to December 2009, between the short and long rainy seasons. There is only one harvest period, leading to seasonal changes in the availability of food. The time of data collection and the four-week recall period occurred four to six months after the main harvest. |

**Supplementary Table 5. Description of food supplementation programs in study community**

| **Country** | **Food Supplementation Programs in Community** |
| --- | --- |
| Bangladesh | The National Nutrition Program runs a food supplementation program for children and pregnant/lactating women of poor SES conditions. The supplement consists of a meal that consists of a mixture of roasted rice powder, roasted lentil powder, mollasses and soybean oil. Each sachet gives 150 kcalories, a child receives 2 sachets while a pregnant women receives 4 sachets daily. However, the program may end this year. Apart from this, the country has several food supplementation programs in areas riddled with severe food insecurity. |
| Brazil | Parque Universitário urban community does not have a regular food supplementation program. |
| India | Children aged 6 months to 3 years are supposed to get a "nutritional mix" [cereal, pulse and oil seed mix]. Children who attend government run crèches [from 3 to 6 years] or government schools are provided a ‘noon meal’ – consisting of rice, dal (lentils) and vegetables, they also get a boiled egg thrice a week. |
| Nepal | None |
| Peru | Households within the community have the option of visiting the local health department to receive a food basket (3 kg of cereal, 1 kg of rice, 1 kg of beans, cooking oil) every month until the child is 1 year old, then every other month until the child is 3 years old. |
| Pakistan | None |
| South Africa | - The National Department of Health provides soft porridge sachets for identified chronically ill patients through clinics. - The National Department of Education provides National School Nutrition Programme. - Daily ‘After School Meals’ for orphanages and children from poor families supported by USAID. Some households also receive food parcels once in a while. |
| Tanzania | None. |
