# Supplementary material for: Household food access and child malnutrition: results from the eight-country MAL-ED study
Source: Popul Health Metr. 2012 Dec 13;10:24. doi: 10.1186/1478-7954-10-24 (PMC3584951; doi:10.1186/1478-7954-10-24)
Supplement: Additional file 2 — Translations of food insecurity questionnaire into Portuguese, Spanish, Sindhi, Swahili and Nepali. [file 1478-7954-10-24-S2.pdf]

Online Supporting Material

PORTUGUESE

QUESTÕES DE SEGURANÇA ALIMENTAR			
1	Nas últimas quatro semanas, você se preocupou que na sua casa não teria alimento suficiente?	Não = 00; Raramente = 01<br>Às vezes = 02; Frequentemente = 03	☐ ☐
2	Nas últimas quatro semanas, você ou algum membro da casa não pode comer algum alimento preferido por falta de recursos?	Não = 00; Raramente = 01<br>Às vezes = 02; Frequentemente = 03	☐ ☐
3	Nas últimas quatro semanas, você ou algum membro da sua casa teve de comer uma variedade limitada de alimentos devido a falta de recursos?	Não = 00; Raramente = 01<br>Às vezes = 02; Frequentemente = 03	☐ ☐
4	Nas últimas quatro semanas, você ou algum membro da sua casa teve de comer algum alimento que realmente não queriam comer devido a falta de recursos para obter outros tipos de alimentos?	Não = 00; Raramente = 01<br>Às vezes = 02; Frequentemente = 03	☐ ☐
5	Nas últimas quatro semanas, você ou algum membro da sua casa teve de comer uma refeição menor do que você sentiu que precisava porque não havia comida suficientente?	Não = 00; Raramente = 01<br>Às vezes = 02; Frequentemente = 03	☐ ☐
6	Nas últimas quatro semanas, você ou algum membro da sua casa teve de fazer menos refeições no dia porque não havia comida suficientente?	Não = 00; Raramente = 01<br>Às vezes = 02; Frequentemente = 03	☐ ☐
7	Nas últimas quatro semanas, faltou algum tipo de alimento na sua casa devido a falta de recursos para comprar?	Não = 00; Raramente = 01<br>Às vezes = 02; Frequentemente = 03	☐ ☐
8	Nas últimas quatro semanas, você ou algum membro da sua casa foram dormir a noite com fome porque não existia alimento suficiente?	Não = 00; Raramente = 01<br>Às vezes = 02; Frequentemente = 03	☐ ☐
9	Nas últimas quatro semanas, você ou algum membro da sua casa ficaram um dia e uma noite inteira sem comer nada, porque não havia comida suficiente?	Não = 00; Raramente = 01<br>Às vezes = 02; Frequentemente = 03	☐ ☐

Online Supporting Material

SPANISH

PREGUNTAS SOBRE SEGURIDAD ALIMENTARIA			
1	¿En las últimas cuatro semanas, ha estado usted preocupada porque en su hogar no tenían suficiente comida?	No = 00; Raramente = 01<br>A veces= 02; A menudo = 03	☐☐
2	¿En las últimas cuatro semanas, usted o algún miembro de su hogar no ha podido comer el tipo de comida de su preferencia por falta de recursos?	No = 00; Raramente = 01<br>A veces= 02; A menudo = 03	☐☐
3	¿En las últimas cuatro semanas, usted o algún miembro de su hogar ha comido una limitada variedad de alimentos por falta de recursos?	No = 00; Raramente = 01<br>A veces= 02; A menudo = 03	☐☐
4	¿En las últimas cuatro semanas, usted o algún miembro de su hogar no ha tenido que comer algunos alimentos que a ustedes realmente no les gusta comer por falta de recursos para obtener otro tipo de alimentos?	No = 00; Raramente = 01<br>A veces= 02; A menudo = 03	☐☐
5	¿En las últimas cuatro semanas, usted o algún miembro de su hogar ha comido menos alimentos de los que usted siente que necesitaba porque no había suficiente comida?	No = 00; Raramente = 01<br>A veces= 02; A menudo = 03	☐☐
6	¿En las últimas cuatro semanas, usted o algún miembro del hogar, se ha saltado alguna comida del día porque no había suficiente comida?	No = 00; Raramente = 01<br>A veces= 02; A menudo = 03	☐☐
7	¿En las últimas cuatro semanas, ha habido alguien en su hogar que no recibiera comida para poder dar a los demás?	No = 00; Raramente = 01<br>A veces= 02; A menudo = 03	☐☐
8	¿En las últimas cuatro semanas, usted o algún miembro de su hogar, se ha ido a dormir por la noche con hambre porque no había suficiente comida?	No = 00; Raramente = 01<br>A veces= 02; A menudo = 03	☐☐
9	¿En las últimas cuatro semanas, usted o algún miembro de su hogar, ha pasado todo el día y noche sin comer nada porque no había suficiente comida?	No = 00; Raramente = 01<br>A veces= 02; A menudo = 03	☐☐

SINDHI

کاڌ خوراک بابت سوال			
**سوال نمبر**	**سوال**	**ڪوڊ**	**جواب**
1	چا گذريل مهيني (چار هفتن) دوران توهان کي اها پريشاني رهي ته توهان وٽ پورو پيٽ پري کائڻ جيترو کاڌو ناهي؟	نه = 00<br>تمام گهڻ = 01<br>ڪڏهن ڪڏهن = 02<br>اڪثر = 03	☐☐
2	چا گذريل مهيني (چار هفتن) دوران توهان يا توهان جي گهر جو ڪو ڀاتي ڪو پسنديد ه / خاص طعام يا کاڌو کائڻ چاهيندو هجي پر پئسي ڏوڪڙ يا وسيلا نه هجڻ ڪري نه کائي سگهيو هجي؟	نه = 00<br>تمام گهٽ = 01<br>ڪڏهن ڪڏهن = 02<br>اڪثر = 03	☐☐
3	چا گذريل مهيني (چار هفتن) دوران پئسي ڏوڪڙ/وسيلن جي نه هجڻ ڪري توهان ساڳئي / چند قسم جا کاڌا کائيندا هجو؟	نه = 00<br>تمام گهڻ = 01<br>ڪڏهن ڪڏهن = 02<br>اڪثر = 03	☐☐
4	چا گذريل مهيني (چار هفتن) دوران توهان يا توهان جي گهر جو ڪو ڀاتي اهڙو کاڌو کائيندو هجي جيڪو حقيقت ۾ توهان کائڻ ئي نه چاهيندا هجو پر پئسي ڏوڪڙ يا وسيلا نه هجڻ ڪري توهان کاڌا نه وٺي سگهندا هجو؟	نه = 00<br>تمام گهڻ = 01<br>ڪڏهن ڪڏهن = 02<br>اڪثر = 03	☐☐
5	چا گذريل مهيني (چار هفتن) دوران توهان يا توهان جي گهر جو ڪو ڀاتي گهٽ کاڌو کائي سگهيو هجي يا جيتري بک هجي اوترو نه کائي سگهيو هجي چا ڪاڻ ته توهان جي گهر ۾ کاڌو پورو نه هيو؟	نه = 00<br>تمام گهڻ = 01<br>ڪڏهن ڪڏهن = 02<br>اڪثر = 03	☐☐
6	چا گذريل مهيني (چار هفتن) دوران کاڌو گهٽ هجڻ ڪري توهان يا توهان جي گهر جو ڪو ڀاتي گهٽ ويلا (ٽائيم) ماني کائي سگهيو هجي؟	نه = 00<br>تمام گهڻ = 01<br>ڪڏهن ڪڏهن = 02<br>اڪثر = 03	☐☐
7	چا گذريل مهيني (چار هفتن) دوران توهان وٽ کائڻ لاءِ ڪجهه به نه هجي چاڪاڻ ته توهان وٽ پئسا ڏوڪڙ وسيلا نه هئا جو توهان ڪجهه کائڻ لاءِ کاڌو خريد ڪري سگهو؟	نه = 00<br>تمام گهڻ = 01<br>ڪڏهن ڪڏهن = 02<br>اڪثر = 03	☐☐
8	چا گذريل مهيني (چار هفتن) دوران گهربل کاڌو نه هجڻ جي ڪري رات جو توهان يا توهان جي گهر جو ڪو ڀاتي بنا کاڌو کائڻ جي بکيو ستو هجي؟	نه = 00<br>تمام گهڻ = 01<br>ڪڏهن ڪڏهن = 02<br>اڪثر = 03	☐☐
9	چا گذريل مهيني (چار هفتن) دوران کاڌو نه هجڻ ڪري توهان يا توهان جي گهر جو ڪو ڀاتي سڄي ڏينهن ۽ رات ۾ ڪجهه به نه کائي سگهيو هجي؟	نه = 00<br>تمام گهڻ = 01<br>ڪڏهن ڪڏهن = 02<br>اڪثر = 03	☐☐

Online Supporting Material

SWAHILI

#	Question	Code	Response
1	Katika kipindi cha wiki nne zilizopita, ulikuwa na wasiwasi kuwa nyumbani kwako hamtakuwa na chakula cha kutosha?	Hapana = 00; Mara chache = 01; Wakati mwingine = 02; Wakati mwingi = 03	☐☐
2	Katika wiki nne zilizopita, je wewe ama mkazi mwingine wa kaya yako hakuweza kula chakula anachopenda kutokana na upungufu wa mahitaji?	Hapana = 00; Mara chache = 01; Wakati mwingine = 02; Mara nyingi = 03	☐☐
3	Katika wiki nne zilizopita, je wewe ama mkazi mwingine wa kaya yako alilazimika kula aina ya chakula ambacho hakuwa amependa kutokana na upungufu wa aina tofauti za chakula ambao ulisababishwa na upungufu wa mahitaji ya kuwa na aina tofauti ya vyakula.	Hapana = 00; Mara chache = 01; Wakati mwingine = 02; Mara nyingi = 03= 03	☐☐
4	Katika wiki nne zilizopita, je wewe ama mkazi mwingine wa kaya yako alikula chakula ambacho hukupenda kula kwa sababu ya upungufu wa mahitaji ya aina nyingine ya chakula?	Hapana = 00; Mara chache = 01; Wakati mwingine = 02; Mara nyingi = 03	☐☐
5	Katika wiki nne zilizopita, je wewe ama mkazi mwingine wa kaya yako alihitajika kula kiasi kidogo cha chakula kulivyo unavyodhani ilihitajika kutokana na kutokuwapo chakula cha kutosha?	Hapana = 00; Mara chache = 01; Wakati mwingine = 02; Mara nyingi = 03= 03	☐☐
6	Katika wiki nne zilizopita, je wewe ama mkazi mwingine wa kaya yako alilazimika kula milo michache katika siku kwa sababu hapakuwa na chakula cha kutosha?	Hapana = 00; Mara chache = 01; Wakati mwingine = 02; Mara nyingi = 03	☐☐
7	Katika wiki nne zilizopita, je kulikuwa hakuna chakula cha aina yoyote cha kula nyumbani kwako kutokana na kutokuwepo mahitaji ya kutengeneza chakula?	Hapana = 00; Mara chache = 01; Wakati mwingine = 02; Mara nyingi = 03= 03	☐☐
8	Katika wiki nne zilizopita, je wewe ama mkazi mwingine wa kaya yako kuna aliyekwenda kulala akiwa na njaa kwa sababu hapakuwa na chakula cha kutosha?	Hapana = 00; Mara chache = 01; Wakati mwingine = 02; Mara nyingi = 03	☐☐
9	Katika wiki nne zilizopita, je wewe ama mkazi mwingine wa kaya yako kuna aliyekaa na njaa mchana na usiku kucha bila kula chochote kwa sababu hapakuwepo chakula cha kutosha?	Hapana = 00; Mara chache = 01; Wakati mwingine = 02; Mara nyingi = 03= 03	☐☐

Online Supporting Material

NEPALI

FOOD SECURITY QUESTIONS			
1	In the past four weeks, did you worry that your household would not have enough food?<br>वितेको ४ हप्तामा, तपाईंको घरमा खानेकुरा पुग्ने छैन भन्ने कुराले तपाईंलाई पिर परेको थियो ?	No = 00; Rarely = 01<br>Sometimes = 02; Often = 03<br>छैन =००; आकल झुकल =०१, कहिलेकाही =०२, प्रायःजसो=०३	☐☐
2	In the past four weeks, were you or any household member not able to eat the kinds of foods you preferred because of a lack of resources?<br>वितेको ४ हप्तामा, तपाईं वा तपाईंको परिवारको कुनै सदस्यले आफूलाई मन परेको खाना स्रोतको कमीले गर्दा खान सक्नुभएको छैन ?	No = 00; Rarely = 01<br>Sometimes = 02; Often = 03<br>छैन =००; आकल झुकल =०१, कहिलेकाही =०२, प्रायःजसो=०३	☐☐
3	In the past four weeks, did you or any household member have to eat a limited variety of foods due to a lack of resources?<br>वितेको ४ हप्तामा, तपाईं वा तपाईंको परिवारको कुनै सदस्यले स्रोतको कमीले गर्दा सिमीत परिकारका खाना मात्र खानुभएको छ ?	No = 00; Rarely = 01<br>Sometimes = 02; Often = 03<br>छैन =००; आकल झुकल =०१, कहिलेकाही =०२, प्रायःजसो=०३	☐☐
4	In the past four weeks, did you or any household member have to eat some foods that you really did not want to eat because of a lack of resources to obtain other types of food?<br>वितेको ४ हप्तामा, तपाईं वा तपाईंको परिवारको कुनै सदस्यले साधन स्रोतको कमीले गर्दा खानै मन नपर्ने खाना खानपरेको छ (मन परेको खाना किन्न नसक्नाले) ?	No = 00; Rarely = 01<br>Sometimes = 02; Often = 03<br>छैन =००; आकल झुकल =०१, कहिलेकाही =०२, प्रायःजसो =०३	☐☐
5	In the past four weeks, did you or any household member have to eat a smaller meal than you felt you needed because there was not enough food?<br>वितेको ४ हप्तामा, तपाईं वा तपाईंको परिवारको कुनै सदस्यले पर्याप्त खानाको कमीले गर्दा आफूले चाहेको भन्दा कम खाना खानुपरेको छ ?	No = 00; Rarely = 01<br>Sometimes = 02; Often = 03<br>छैन =००; आकल झुकल =०१, कहिलेकाही =०२, प्रायःजसो=०३	☐☐
6	In the past four weeks, did you or any other household member have to eat fewer meals in a day because there was not enough food?<br>वितेको ४ हप्तामा, तपाईं वा तपाईंको परिवारको कुनै सदस्यले पर्याप्त खानाको कमीले दिनमा थोरै पटक खाना खान' परेको छ ?	No = 00; Rarely = 01<br>Sometimes = 02; Often = 03<br>छैन =००; आकल झुकल =०१, कहिलेकाही =०२, प्रायःजसो=०३	☐☐
7	In the past four weeks, was there ever no food to eat of any kind in your household because of lack of resources to get food?<br>वितेको ४ हप्तामा, साधन स्रोतको कमीले गर्दा तपाईंको घरमा कुनै पनि खाना नभएको अवस्था थियो ?	No = 00; Rarely = 01<br>Sometimes = 02; Often = 03<br>छैन =००; आकल झुकल =०१, कहिलेकाही =०२, प्रायःजसो =०३	☐☐
8	In the past four weeks, did you or any household member go to sleep at night hungry because there was not enough food?	No = 00; Rarely = 01<br>Sometimes = 02; Often = 03<br>छैन =००; आकल झुकल =०१, कहिलेकाही =०२, प्रायःजसो =०३	☐☐

	वितेको ४ हप्तामा, तपाईं वा तपाईंको परिवारको कुनै सदस्य पर्याप्त खानेकुरा नभएकोले राती भोकै सुत्नुपरेको छ ?		
9	In the past four weeks, did you or any household member go a whole day and night without eating anything because there was not enough food?<br>वितेको ४ हप्तामा, तपाईं वा तपाईंको परिवारको कुनै सदस्य पर्याप्त खानेकुरा नभएकोले दिनभर र राती भोकै सुत्नुपरेको छ ?	No = 00; Rarely = 01<br>Sometimes = 02; Often = 03<br>छैन =००; आकल झुकल =०१, कहिलेकाही =०२, प्रायःजसो =०३	☐☐
